# Forward Osmosis in India: Status and Comparison with Other Desalination Technologies

**DOI:** 10.1155/2014/175464

**Published:** 2014-10-29

**Authors:** Dhruv Mehta, Lovleen Gupta, Rijul Dhingra

**Affiliations:** Department of Environmental Engineering, Delhi Technological University, Shahbad Daulatpur, Main Bawana Road, Delhi 110042, India

## Abstract

With an increase in demand of freshwater and depleting water sources, it is imperative to switch to seawater as a regular source of water supply. However, due to the high total dissolved solid content, it has to be desalinated to make it drinkable. While desalination technologies have been used for many years, mass deployment of such technologies poses a number of challenges like high energy requirements as well as high negative environmental impact through side products and CO_2_ emissions. The purpose of this paper is to present a sustainable technology for desalination. Forward osmosis, an emerging technology, is compared with the other commonly used technologies worldwide, namely, multieffect distillation, multistage flash distillation, and reverse osmosis as well as other emerging technologies like vapour compression, solar humidification dehumidification, nanofiltration, and freezing desalination. As energy consumption and associated greenhouse gas emissions are one of the major concerns of desalination, this paper concludes that forward osmosis is an emerging sustainable technology for seawater desalination. This paper then presents the challenges involved in the application of forward osmosis in India and presents a plant setup. In the end, the cost comparison of a forward osmosis and reverse osmosis plant has been done and it was concluded that forward osmosis is economically better as well.

## 1. Introduction

Three-fourths of the earth's surface is covered with water. Out of this three-fourths, only 0.6 percent is found in the form of lakes and rivers [1]. The rest is found in the form of salt water in oceans, which cannot be directly used for human consumption. The world's population is expected to reach 8.1 billion by the end of 2025 [2]. With depleting fresh water sources, additional water supplies will be important in determining the future of humans on this planet. Like fossil fuel resources, the need for water is likely to be a major factor for world stability in the coming few years [3, 4]. There are technological innovations being made, as well as new water sources being explored, to cater to the growing water demand around the world. If used efficiently, the concept of desalination is a very viable option for addressing the problem of water scarcity.

Desalination is slowly becoming a popular concept among the public. The worldwide desalination industry is expected to grow 140 percent over the next decade, with 25 billion United States Dollar (USD) in capital investment by 2010 and 56 billion USD by 2015 [5]. Thermal methods of desalination such as multistage flash (MSF) and multiple-effect distillation (MED) have been found successful in converting seawater into pure water, but the energy required for this process makes it unviable to be used on a large scale. Membrane technology, such as reverse osmosis (RO), suffers from membrane fouling and a high rate of brine production [6]. Current methods of brine disposal cause major environmental problems. Therefore, research is continually being carried out in order to find out the sustainable way of desalting seawater. One such method is forward osmosis (FO). It is found to be highly efficient with a low rate of brine production; FO is an area of active research. If adopted, this technique might be the answer to all of our water problems.

India has a coastline of 7516 km [7]. Since, 9 Indian states and 4 union territories are surrounded by seawater along one or more sides, desalination can play a major role in augmenting the fresh water requirements of the country if technically and economically efficient desalination technology is implemented for obtaining pure water from sea water in these regions. Additionally, areas such as Tamil Nadu, Gujarat, and Andhra Pradesh have a huge problem of water scarcity due to poor river water availability, low ground water levels, and high water demand [8]. Since the launch of the first five-year plan in 1951, 1105 billion Indian Rupees [9] have been spent to provide safe drinking water, yet it continues to be a major issue. Thus, it is imperative that India focuses its attention on seawater in order to meet the water demands of the growing population.

Of all the desalination techniques available, in this paper, we focus our attention on FO. The review begins by discussing the current situation of the desalination sector in India. The key part of this paper is the comparison of FO with all of the other prevalent, as well as emerging, techniques in India. We also cite all the benefits of adopting FO. Then we discuss the application of FO in seawater desalination along with its advantages and disadvantages. In the final part of the review, we look at the challenges of bringing FO into the Indian market. Due to limitations on literature on the topic, a complete comparison of the techniques on all 14 parameters could not be made.

## 2. Desalination in India

**Figure 1:**
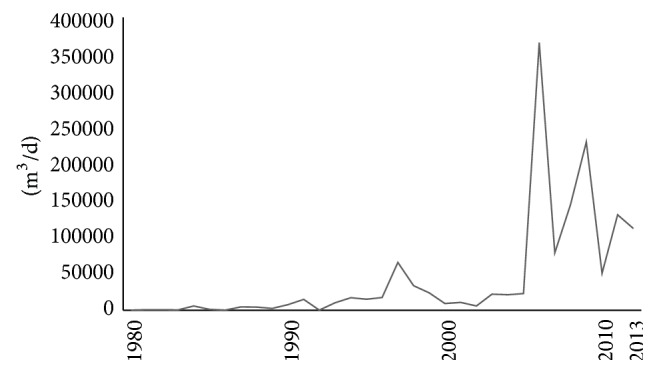
Increase in capacity of plants desalinating saline water in India (DesalData).

India represents 16 percent of the world's population and at its present growth rate is expected to hit the 1.5 billion mark within the next four decades [10]. The Government of India's Planning Commission estimates the water demand to increase to 1180 BCM (billion cubic meters) by the year 2050. This represents a nearly 66 percent increase from the known 710 BCM of water demand in 2010 [11]. With the depleting water sources and increase in the demand, it becomes imperative to switch to seawater to meet the water requirements in India. Suryanarayanan says that desalination technologies are still evolving in India [12] and Indo-Asian News Service report suggests that the Indian desalination water market is expected to grow at a compounded annual growth rate (CAGR) of 30 percent over a period of five years from 2013 to 2018 [13]. There has been a sudden increase in the capacity of plants desalinating saline water from year 2006 as shown in Figure 1. In addition, the desalination capacity in India is seen at 5.35 million m^3^/d by 2018 [13], which is 342.1% increase from its capacity in 2013. Thus, it can be concluded that desalination water market in India is expected to grow at a rapid rate.

**Figure 2:**
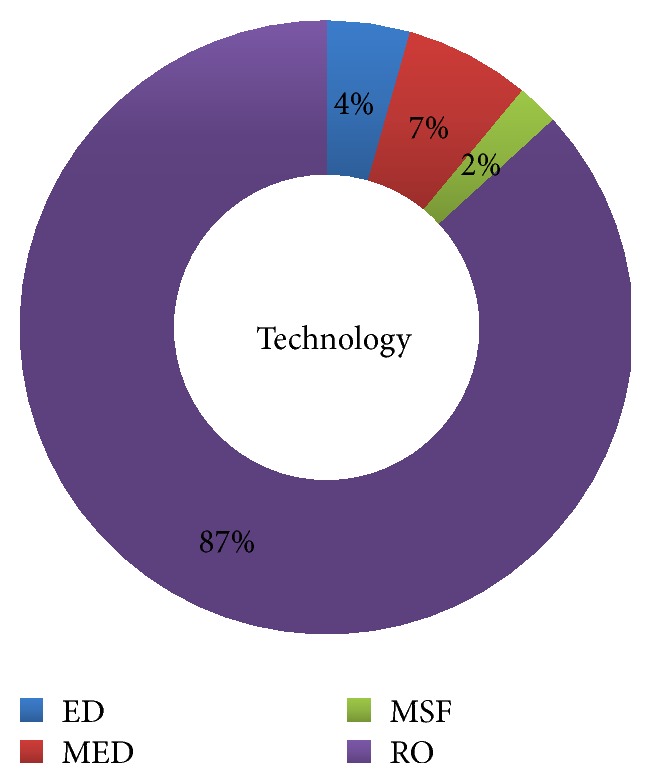
Desalination technologies adopted in India in 2012-13 (DesalData).

The two main desalination technologies are those based on thermal and membrane processes. Thermal processes include multistage flash (MSF), multiple-effect distillation (MED), and vapour compression (VC), whereas membrane processes include reverse osmosis (RO), electrodialysis (ED), and forward osmosis (FO).  Figure 2 shows the distribution of the desalination technologies adopted in India in the financial year 2012-13. Since membrane based technologies are considered to be 23 percent cheaper than thermal based technologies for generating desalinated water, plants in India are mostly membrane based [14]. It can be seen that RO dominated.

**Figure 3:**
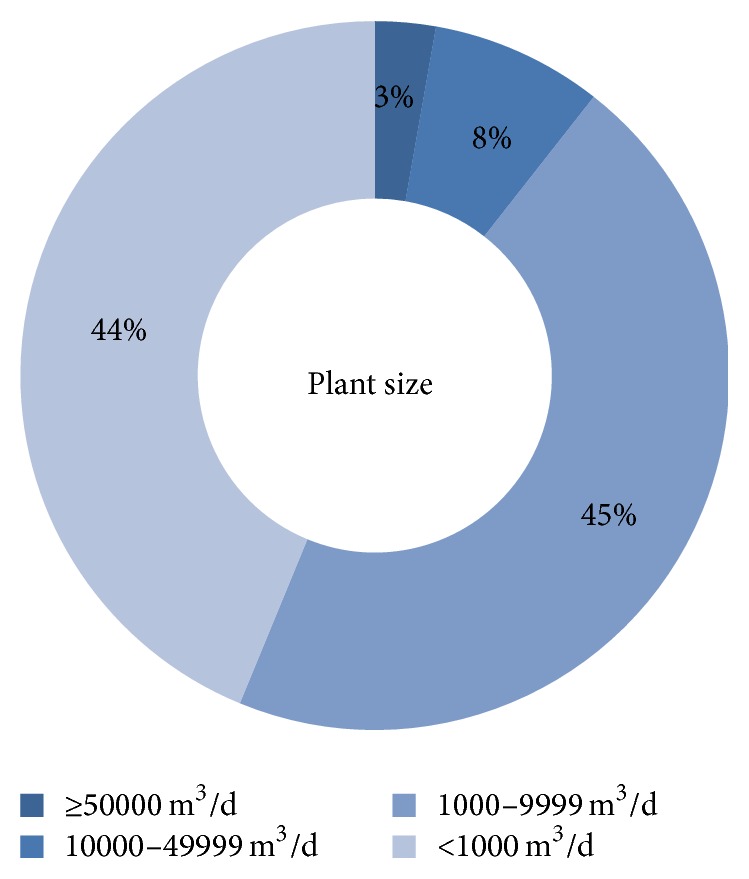
Plant size breakup in India in 2012-13 (DesalData).

Figure 3 illustrates the breakup of the desalination plants currently operating in India by capacity. It can be seen that currently desalination plants in India are mostly of sizes up to 10,000 cubic meters per day (m^3^/d). These plants are mostly used for industrial and irrigational purposes. Gujarat and Tamil Nadu have emerged as desalination hubs due to poor water availability, low ground water levels, and high demand [8]. Desalination is also used in the coastal regions of Maharashtra, Andhra Pradesh, Karnataka, and West Bengal.

## 3. Desalination Techniques

### 3.1. Current Techniques Employed in India

#### 3.1.1. Reverse Osmosis

Reverse osmosis (RO) is a membrane separation process in which the seawater permeates through a membrane by applying a pressure larger than the osmotic pressure of the seawater. The membrane is permeable for water but not for the dissolved salts. In this way, a separation between a pure water fraction (the product) and a concentrated fraction (the retentate or concentrate) is obtained [15]. A typical RO desalting plant consists of three sections, namely, pretreatment section, membrane section, and posttreatment section. Energy consumption depends on the salt content of the feed water. Development of RO membranes of very high rejection, while maintaining high permeability, has the potential to reduce energy consumption [16].

Bhabha Atomic Research Centre erected desalination plants in Andhra Pradesh, Gujarat, Rajasthan, Tamil Nadu, Port Blair, and Lakshadweep. Bharat Heavy Electricals Limited (BHEL) operated 12 desalination plants using RO technology in Tamil Nadu [17].

#### 3.1.2. Multistage Flash

Multistage flash (MSF) starts with saline feed water being passed through a series of tubes which preheat the water. Water then enters the brine heater where it is heated using thermal energy. The heated water is then passed through a vessel, called the stage, with pressure lower than that of the brine heater, which results in flashing of the saline water. Vapours formed during boiling condense on the tubes carrying the input saline water. Distillate is collected in the distillate trays. Only a small percentage of the heated water is converted into steam, depending upon the pressure maintained at the next stage. The remaining water is then introduced to a subsequent stage, which has a lower pressure. This process continues until the saline water is cooled down and discharged [18].

BARC built a 425 KLD MSF pilot plant in Bombay and a 4.5 MLD MSF unit as a part of 6.3 MLD Hybrid MSF-RO plants [16].

#### 3.1.3. Multieffect Distillation

Multieffect distillation (MED) takes place in a series of vessels or effects and uses the principle of evaporation and condensation at reduced ambient pressures. The feed water, after being preheated in the final condenser, is fed in equal proportions to the various vessels. The water is sprayed on the evaporator surface (tubes) after being heated to its boiling point. The evaporator tubes in the first effect are heated with steam. The steam comes from the steam turbines of a power plant, or boiler, where the steam inside condenses as the water sprayed on the evaporators vaporizes. The vapours from the first effect are used to heat the surfaces of the succeeding effects. The vapours produced in the last effect are condensed in the final condenser—cooled by incoming saline feed water—to yield pure water [18].

At reliance industries ltd. (RIL) in India, four MED plants have been in operation since 1998, each with a nominal production of 12,000 m^3^/d. A fifth MED unit of 14,400 m^3^/d capacity was operational in February 2005 [19].

### 3.2. Other Techniques Being Researched

#### 3.2.1. Vapour Compression

Vapour compression (VC) is a highly reliable and efficient process which uses mechanical energy to desalinate water. Heat is pumped from a reservoir which is at a low temperature to a reservoir which is at higher temperature. The basic system consists of an evaporator-condenser and a compressor where water vapours act as a working fluid. Water vapour at the evaporation side of a heat transfer surface is compressed. This results in a rise of the saturation temperature and pressure, which is then condensed to form condensate releasing latent heat of condensation in the process. The latent heat is transferred across the heat transfer surface into the water layer side causing further evaporation.

#### 3.2.2. Solar Humidification-Dehumidification

In solar humidification-dehumidification (SHDH), solar energy is used to heat sea water, which results in the humidification of air. This air acts as a carrier gas. The humidified air rises up to form clouds, which upon dehumidification pours down as rain. The man-made version of this cycle is called the humidification-dehumidification desalination cycle.

#### 3.2.3. Electrodialysis Reversal

The electrodialysis reversal (EDR) process is based on the principle that most dissolved salts are positively and negatively charged and are attracted towards an electrode of the opposite charge. Selective membranes allow either passage of anions or cations, leading to the separation of ions. Anion selective membranes allow the passage of anions but block cations, whereas cation selective membranes allow the passage of cations but block anions. Polarity of the electrode is frequently reversed to free up accumulated charge on the membrane surface, thus minimizing membrane fouling.

#### 3.2.4. Freezing Desalination

The process of freezing desalination (FD) is based on the principle that the formation of ice crystals leads to the exclusion of dissolved solids. During freezing, the nonfrozen saline component is removed at the appropriate time. The frozen water is rinsed to remove the remaining salts adhering to the ice crystals. The ice is then melted to get fresh water [20].

#### 3.2.5. Nanofiltration

In nanofiltration (NF), a pressure driven membrane separation process, the separation takes place across a semipermeable membrane. Membranes have pores that measure in the nanometers. The pressure difference between the feed and filtrate side of membrane leads to the separation of salt from water. Lower operation pressure, higher flux, and higher retention of multivalent anion salts provide it an advantage over RO.

#### 3.2.6. Forward Osmosis

Forward osmosis (FO) is a natural phenomenon where a difference in osmotic pressure drives the solvent from a region of lower concentration to a region of higher concentration across a selectively permeable membrane [21]. The low-concentration solution is called the feed solution and the high-concentration solution is called the draw solution [22]. Feed solution is generally seawater or brackish water that needs to be desalinated, while the draw solution is prepared in a laboratory using a solute of high osmotic pressure. Osmotic pressure difference between the feed and draw solution causes the water to flow into the draw solution from the feed solution [23]. Energy efficient techniques are used to draw pure water from the draw solution [24]. Regeneration of the draw solution is the most expensive stage in the FO desalination process. Nanofiltration, reverse osmosis, or other thermal processes are proposed for the regeneration of draw solution [25].

## 4. Comparison between the Desalination Technologies

Table 1 provides a detailed comparison of the traditional techniques as well as techniques being researched for desalination. It can be seen that FO requires less energy per m^3^ of water than the other thermal and membrane techniques. Solar energy is in abundance, but the technical knowledge to harness it efficiently is still primitive. Nanofilters have also shown early promise but there is still time before its theoretical knowledge can be efficiently applied to desalination. Stand-alone nanofilters face many problems, such as fouling, and therefore need to be incorporated with another suitable technique as a hybrid system. The cost required to construct an FO plant is considerably less than most of the other thermal technologies, even reverse osmosis. Compared to RO, FO has less membrane fouling, scaling, and brine discharge. Even though the product water quality varies over a wider range than the thermal methods, this is not of much importance since the dissolved solid content is well under the specified world health organization (WHO) guidelines. Greenhouse gas (GHG) emissions are also considerably less in membrane techniques compared to the thermal techniques. However, the draw solutions and membranes in FO are still areas of active research in order to improve upon the process. Additionally, though FO membranes suffer from fouling which other techniques besides membrane techniques do not, the advantages of FO outweigh its disadvantages. Therefore, if efficiently used, FO can prove to be an efficient way of desalinating seawater.

## 5. Challenges in Settingup of FO Plant

- Membrane development: an appropriate membrane should be able to reduce the effect of concentration polarization, fouling, and reverse solute diffusion. Values of FO flux calculated using solution diffusion theory tend to have higher values than experimental values. This is attributed to the presence of internal and external concentration polarization which reduces the osmotic force.
- Draw solution development: a suitable draw solution should have high osmotic pressure, be easily recoverable, and exhibit minimal internal concentration polarization. Factors such as low cost, zero toxicity, and low fouling are also important.
- Membrane fouling: membranes having low fouling are still being researched. Low membrane fouling would lead to better quality product water. Membranes would have a longer working life leading to reduction in capital and operational costs.
- Brine discharge: disposal of brine produced is a major issue since it causes environmental problems. However, not only is it a challenge for FO, but also it is a challenge for every other desalination technology.
- Fragmentation: creating an industrial standard would be a problem because most of the Indian market is fragmented with many regional players [26].
- Bureaucratic hurdles: new technology adopted has to be approved by the government before it can be introduced into the market. Slow execution and bureaucratic hurdles may prove to be time consuming and cause problems before the technology can be adopted [26].
- Improper training: as the technology is new, lack of adequate training of the staff handling the plant would be a major problem for the industry.

## 6. Proposed FO Setup in India

**Figure 4:**
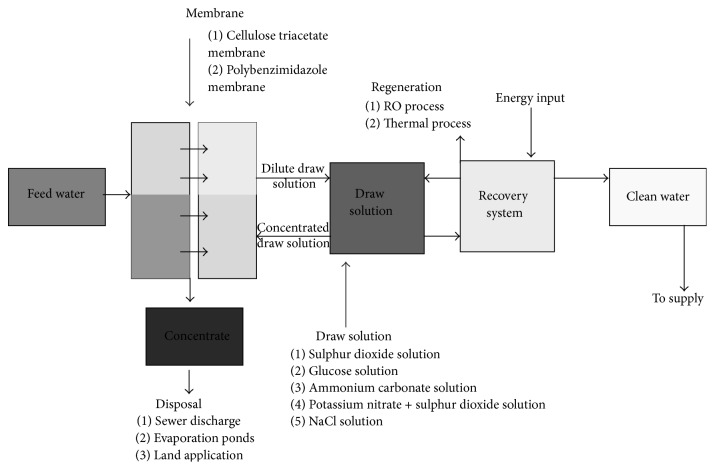
Proposed FO setup viable for use in the Indian market.

Figure 4 presents the FO setup as proposed for India. 50 leading scientists and professionals working in the field of desalination in India were surveyed on the potential FO setup that can be viably operated in the Indian market. According to the results of the survey, the FO membranes which can be easily synthesized using the existing setup in India are (a) cellulose triacetate membrane and (b) polybenzimidazole. The possible draw solutions which can be used in the Indian market are (a) sulphur dioxide solution, (b) glucose solution, (c) ammonium carbonate solution, (d) sodium chloride solution, and (e) potassium nitrate + sulphur dioxide solution. Also, for regeneration purposes, RO or any thermal process can be employed, as the technical knowhow of these processes is very good in India considering the data presented in Figures 2 and 3. It is proposed that the concentrate from the process is disposed of using any of the following three techniques independently or in combination of each other: (a) sewer discharge, (b) evaporation ponds, and (c) land application.

## 7. Cost Analysis

The costs of desalination vary significantly depending on the size and type of the desalination plant, the source and quality of incoming feed water, the plant location, site conditions, qualified labour, energy costs, and plant lifetime. Lower feed water salinity requires less power consumption and dosing of antiscale chemicals. Larger plant capacity reduces the unit cost of water due to economies of scale. Lower energy costs and longer plant period reduce unit product water cost [27].

The primary elements of desalination costs are capital cost and annual running cost. The capital cost includes the purchase cost of major equipment, auxiliary equipment, land, construction, management overheads, and contingency costs. Annual running costs consist of costs for energy, labour, chemicals, consumables, and spare parts [28].

As is evident from Figure 2, approximately 88% of the desalination plants operating in India are based on RO, and the cost analysis of RO and FO has been done. We have used the model developed by CH2M Hill (a global environmental consulting company), which estimates the cost involved in setting up an FO plant and RO plant [29]. The model has been found useful in estimating costs involved up to 15 mgd capacity of recovered water from impaired solution.

The important assumptions on which cost model of both SWRO and FO depends are [29]

- number of hours per day the plant operates—24 hours,
- number of days per year the plant operates—329 days,
- membrane replacement frequency—6 years,
- number of membrane replacements in 20 years—3.

Membrane replacement costs, cartridge filter replacement costs, labour, and chemical costs are included in the Annual Operation and Maintenance cost in both SWRO and FO models.

Table 2 shows the estimated costs involved in setting up both FO and RO plants of design capacity 1 mgd.

A plant operating on FO technology can be constructed at 90% of the construction cost and operated at 80% of the operation cost of an SWRO plant, with the current options of FO membranes and draw solution available. Extensive research is being carried out to develop FO membranes offering better flux performance and draw solutions which can be much more easily regenerated. As and when better membranes and draw solutions are available, the construction and operating cost of FO plants is expected to reduce further.

## 8. Conclusion

Most of the present desalination techniques are currently areas of active research, which is being performed to constantly find ways of improving the technologies and reducing the capital cost as well as increasing the efficiency. Early signs show FO to be the most promising technique that can be used efficiently to meet India's water demand. FO requires low capital input and results in lesser membrane fouling, and the product water is of high quality. Low capital cost is going to act as an incentive, resulting in increased expenditure for the adoption of this technique. However, lack of trained personnel will prove to be a hindrance and so there is still time before this technique proves to be a major player in the Indian market.

## Figures and Tables

**Table 1:** Comparison of different desalination techniques.

Desalination technique	MSF	MED	RO	VC	SHDH	EDR	FD	NF	FO
Energy consumption (kWh/m^3^)	Electrical: 4–6 Thermal: 55–120 [30]	Electrical: 2–2.5 Thermal: 30–120 [31]	Electrical: 3.5–4.5 Thermal: None [30]	Electrical: 7–12 Thermal: None [32]	140–550 [33]	<0.6 [34]	90–120 [35]	0.3–1 [36]	Total: 0.84 [37]

Product water quality (mg/L TDS)	1–50 [30]	1–50 [38]	1–350 [30]	1–50 [39]	50–90 [40]	0–10 [41]	Not available	30–45 [42]	120–200 [43]

Total capital costs	Very high	High	Low-medium	Medium	Very high	Low-high	Very high	Low	Low

Tolerance to changes in seawater composition	Medium-high	High	Very low	High	Very high	Very high	high	Very low	Very low

Maintenance requirements	Medium	Low	High	High	Higher than RO	High	Very high	low	High but with less frequent cleaning than RO

Scaling potential	Low-medium	Low	High	Medium	Low	Very low	Very low	Less than RO	High but lower than RO

Chemical consumption	High	Medium	High	Medium	—	Low	Low	Low	Medium

Ratio between product to total seawater flow	0.1-0.2 [30]	0.1–0.25 [30]	0.3–0.5 [30]	0.7 [44]	0.85 [45]	0.8-0.9 [41]	0-1 [46]	0.7 [47]	0.3–0.85 [48]

Technical know how	High	High-medium	Medium	Medium	Low	Medium	Medium	Low	Low

Membrane fouling	NA	NA	High	NA	NA	Low	NA	High	Low

GHG emission	Yes	Yes	Negligible	Yes	No emission	Can't say	Can't say	Negligible	Negligible

Fully automatic operation	Possible	Possible	Possible	Possible	No information available	Possible	No information available	Possible	Possible

Limiting factors	Pumps, valves, and vacuum unit [30]	Erection and construction aspects; plant reliability [30]	Pumps [30]	Volumes of vapour to be handled are large, thus demanding high volumetric capacity centrifugal compressors. [44]	Low productivity and thermal efficiency, and large land area required [49]	Microbial contamination [34]	High capital cost and operating cost, and retention of aroma from original water [50]	Membrane fouling, limited lifetime of membranes, and low chemical resistance [51]	Lack of an optimized membrane to produce high water flux, and lack of ideal draw solution [52]

**Table 2:** Cost comparison between RO and FO seawater desalination plant.

Seawater reverse osmosis	Forward osmosis
Design capacity = 1 mgd Construction cost (in USD) = −39877 ∗ *C* ^2^ + 3*E* + 06 ∗ *C* + 7*E* + 06, where *C* is the design capacity in mgd [29] = −38977 ∗ (1) + 3*E* + 06 + 7*E* + 06 = **USD 9.96 million** Annual operation and maintenance cost (in USD) = 697283 ∗ *C* + 368927, where *C* is the design capacity in mgd [29] = 697283 + 368927 = **USD 1.06 million**	Design capacity = 1 mgd Construction cost (in USD) = 86.82 (*A*) + 3*E* + 06, where *A* is membrane area in sq ft. [29] FO membrane area required = design capacity/water flux performance Water flux performance = 15 gfd [53]. This value is for a pilot plant developed by Yale University which used ammonia-carbon dioxide as draw solution and cellulose acetate membrane. Thus, FO membrane area required (*A*) = 10^6^/15 *A* = 66666.6 sq ft. Thus, construction cost = 86.82 (66666.6) + 3*E* + 06 = **USD 8.78 million** Annual operation and maintenance cost (in USD) = 3*E* − 06 ∗ *A* ^2^ + 10.631 ∗ *A* + 116981, where *A* is membrane area in sq ft. [29] = 3*E* − 06 ∗ (66666.6)^2^ + 10.631 ∗ 66666.6 + 116981 = **USD 0.83 million**
